# Aborted sudden cardiac death due to ventricular fibrillation in a female patient with mitral valve prolapse

**DOI:** 10.1186/s12947-020-00236-9

**Published:** 2021-01-27

**Authors:** Sofien Ayed, Rainer Hoffmann

**Affiliations:** Department of Cardiology, Angiology and Sleep Medicine, Bonifatius Hospital Lingen, Wilhelmstrasse 13, 49808 Lingen, Germany

**Keywords:** Mitral valve prolapse, Sudden cardiac death, Risk marker

## Abstract

**Background:**

Mitral valve prolapse is the most frequent valvular defect associated with a wide range of electro-hemodynamic abnormalities, leading to heart failure, arrhythmias and sudden cardiac death. Mitral valve prolapse, first described from Barlow in the 1960s, is defined as displacement of mitral leaflet tissue into the left atrium past the mitral annular plane during systole. The correlation between mitral valve prolapse and sudden cardiac death has been investigated and clarified by various studies in recent years. However, identifying patients at risk and applying measures to prevent those from sudden cardiac death is challenging.

**Case presentation:**

We report on a 61-year-old female patient who had undergone an aborted sudden cardiac death. An arrythmogenic mitral valve prolapse was diagnosed. In addition, electrocardiographically and morphologically risk markers for sudden cardiac death were found in this case. We performed an ICD implantation as secondary prophylaxis and intended to reconstruct the mitral valve.

**Conclusion:**

This article examines the association of mitral valve prolapse with sudden cardiac death, the underlying pathophysiological mechanisms and the strategies leading to identify the risk group.

## Introduction

**Fig. 1:**
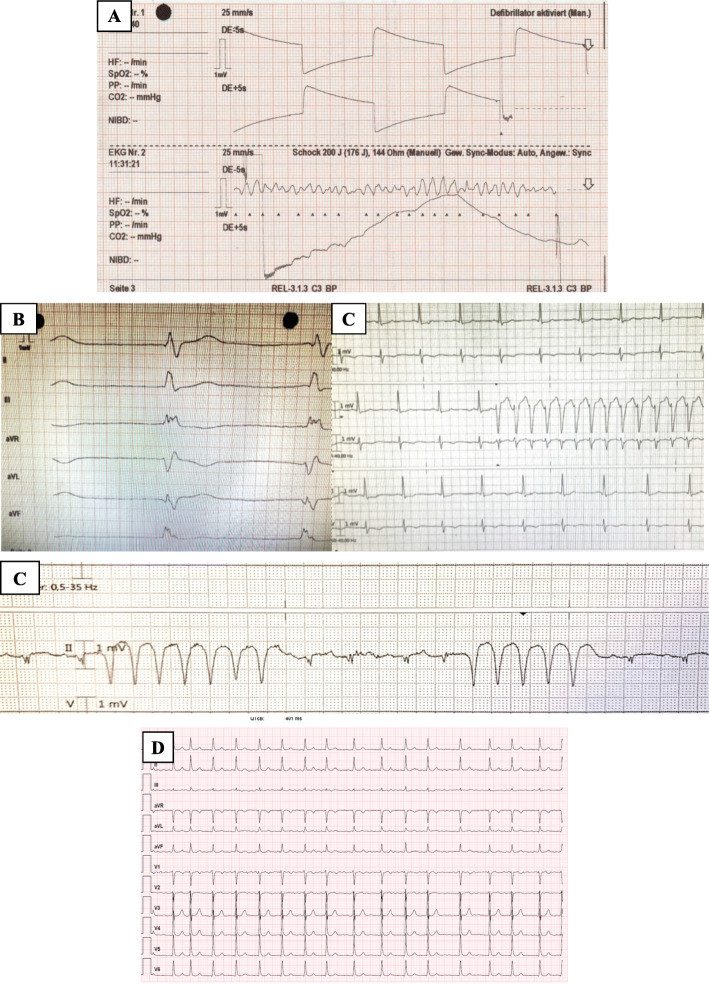
Ventricular fibrillation being terminated with an external defibrillation (**a**). T inversion in the inferior leads (III, aVF) (**b**). Episodes of ventricular arrhythmia detected during monitoring(**c**). Atrial fibrillation with tachycardia (**d**)

**Fig. 2:**
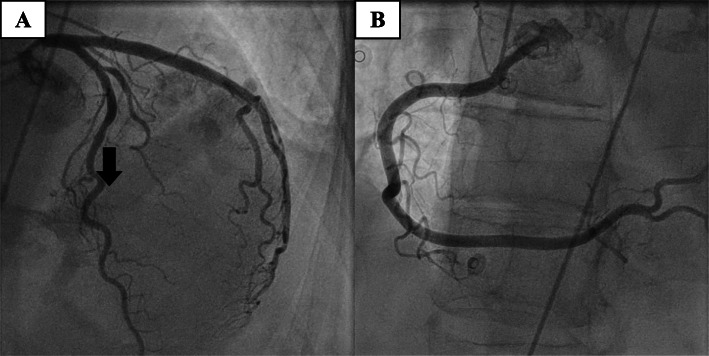
Coronaroangiographie demonstrating normal coronary artery

We report on a patient who had undergone out-of-hospital cardiopulmonary resuscitation. The 61 years old female patient was without any significant previous illness and did not take any medication regularly. The collapse was preceded by malaise and nausea for 10 min. The patient’s partner started cardiopulmonary resuscitation after calling the rescue service. The emergency physician diagnosed ventricular fibrillation (Fig. 1a), which was defibrillated 6 times in total. After 20 min spontaneous circulation was restored. Immediate transfer to our cardiac catheterization laboratory took place. Coronary artery disease was ruled out invasively (Fig. 2). High-sensitivity troponin was repeatedly negative. Pulmonary embolism and subarachnoid hemorrhage were ruled out by means of CT. The 12-lead ECG showed negative T-waves in the inferior leads (Fig. 1b). Thus, no obvious cause for the ventricular fibrillation could be determined initially.

## Case presentation

### Initial diagnostic findings

- Physical examination on admission: Obese 61-year-old woman (BMI 30.1 kg/m2), pulse 60/min, blood pressure 120/80 mmHg, temperature 36 °C, O2 saturation 94% under mechanical ventilation. Heart, lungs, and abdomen unremarkable.
- Laboratory studies: Complete blood count: unremarkable. Plasma coagulation study: normal, D-dimers within normal limits, Clinical chemistry: C-reactive protein 6 mg/L (normal 5 mg/L), all other values within normal limits.
- ECG: Sinus rhythm, HF 60, no AV-Block, no QT time prolongation, inverted T-waves in the inferior leads (III, aVF) (Fig. 1b).
- Fast echocardiography: Normal systolic right and left ventricular systolic function, severe mitral regurgitation, no pericardial effusion.
- Chest x-ray: Normal cardiac size with evidence of pulmonary venous congestion.

### Initial therapy and further diagnostic work-up

Intensive care therapy achieved stabilization of the patient’s general condition. Controlled ventilation was stopped on the fourth day. Except for a hypoactive delirium, which was adequately controlled by administration of a neuroleptikum, no neurological deficits were manifested.

Evaluation of the 12-lead ECG did not reveal any evidence of ECG changes, which are normally associated with canalopathies (Brugada, ARVC, long-, short QT syndromes etc.).

However, we have registered recurrent ventricular salves during monitoring (Fig. 1c). In addition, we registered a short-lasting atrial fibrillation with spontaneous termination (Fig. 1d). Therefore, oral anticoagulation was initiated.

**Fig. 3:**
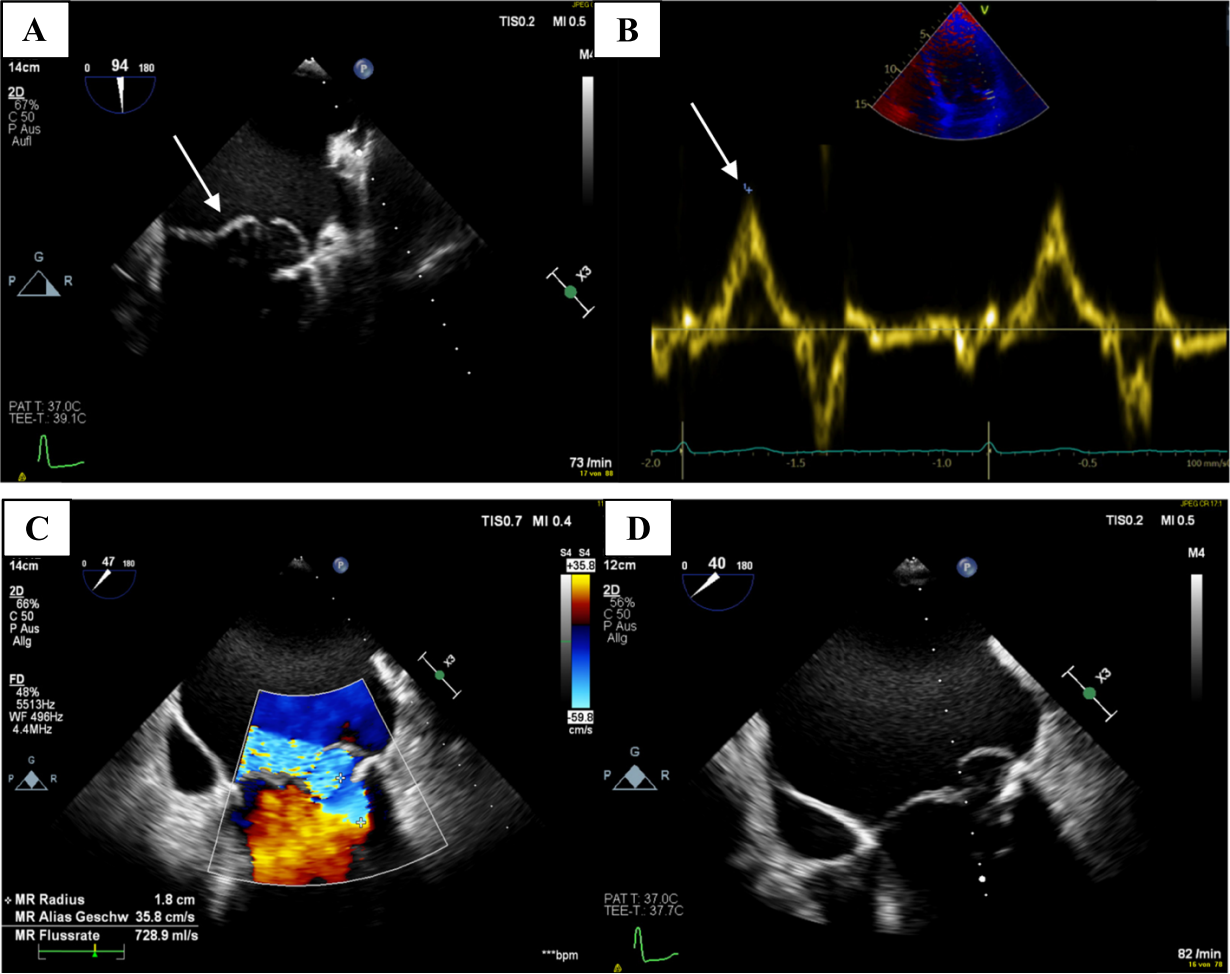
TEE demonstrating bileaflet mitral valve prolapse (**a**). High-velocity mid-systolic spike (lateral annulus, 18 cm/s) (**b**). Severe eccentric mitral regurgitation in TEE (**c**). Dilated left atrium and myxomatous degeneration of the mitral valve (**d**)

Follow-up echocardiography showed a significant mitral valve insufficiency with preserved left ventricular pump function. The left ventricle demonstrated mild endsystolic and enddiastolic dilatation. A severe dilatation of the left atrium (LA area 45 cm2) was noticed (Fig. 3a). The lateral mitral annular velocities was quantified with Doppler tissue imaging. The peak systolic lateral mitral annulus velocity was 18 cm/s (Fig. 3b).

Transesophageal echocardiography was performed to evaluate the mitral valve more precisely. A high-grade eccentric mitral valve insufficiency due to a pronounced PML and a mild AML prolapse could be demonstrated (Fig. 3c). Especially the PML showed thickening and myxomatous changes (Fig. 3d). The tendinous chordae appeared to be intact.

**Fig. 4:**
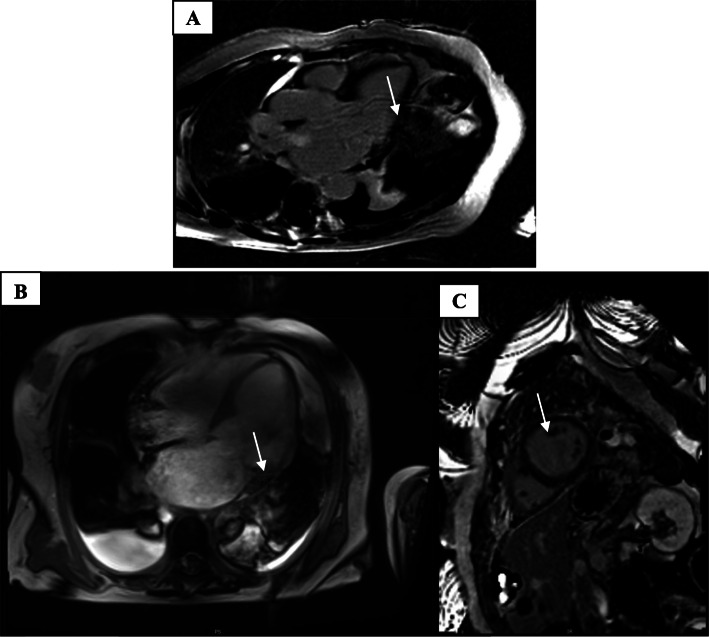
CMR demonstrating LGE posterobasal (**a**). LGE identified in papillary muscle (**b**). Significant MAD 11,2 mm in CMR (**c**)

For further clarification of the arrhythmogenic event, we ordered a CMR. This exploration has shown a discreet mid-wall LGE in the LV inferobasal region (Fig. 4a). Additionally, LGE of the PM was visible on mid short-axis view (Fig. 4b). The right ventricle showed no pathology by echocardiography and CMR. A significant mitral annulus disjunction (MAD) measuring 11,2 mm was identified. (Fig. 4c).

Regarding the severe mitral valvular insufficiency due to the MVP, the case was discussed in the heart team. It was decided to provide the patient with an internal automatic cardioverter defibrillator (ICD) first and to repair the mitral valve by mini invasive surgery in 3 months.

**Fig. 5:**
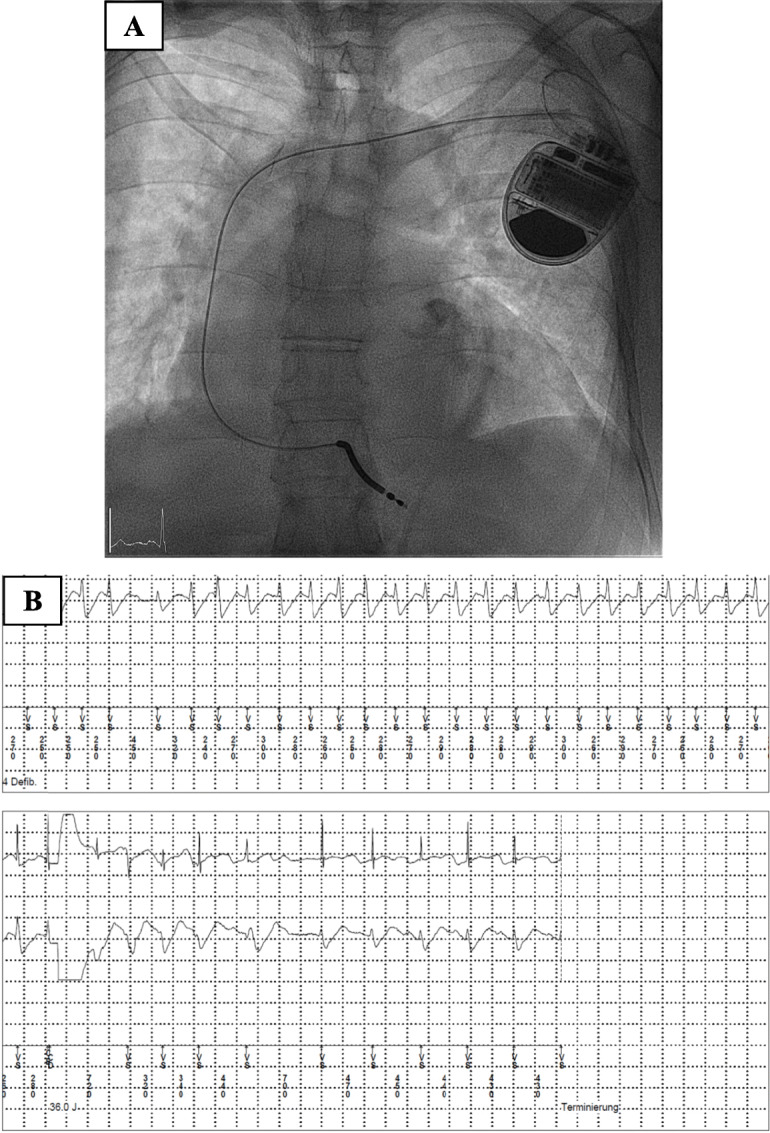
X-Ray showing a single-chamber ICD (**a**). Detection of a ventricular tachycardia and termination with an affectiv shock (**b**)

The patient showed regression of the delirium and an increasing mobility. After implantation of an ICD device (Fig. 5a), the patient was discharged in good general condition and without significant neurological deficits. A cardio-neurological rehabilitation was organized. The first ICD follow-up analysis after 3 Weeks did not show any arrhythmogenic events. At the 3 months ICD follow up, few days after the mitral valve reconstruction, we detected a VT, which has been successfully electrocardioverted (Fig. 5b).

## Discussion

MVP is a relatively common cardiac valvular disorder, affecting about 1–3% of the population. Characteristics include superior displacement of mitral valve leaflet(s) during systole by at least 2 mm, myxomatous degeneration, and thickening of the leaflets [1].

A clinical-pathological investigation performed in Italy, which lasted 21 years and included young persons (< 35 years of age) MVP dying suddenly, has found that the third most common cardiac condition associated with SCD with an incidence of 12% is due to an “arrhythmogenic MVP”, succeeding arrhythmogenic right ventricular dysplasia (24%) and atherosclerotic coronary artery disease (20%) [2].

Two factors are considered fundamental for the occurrence of SCD in patients with MVP: LV myocardial fibrosis (substrate) and complex ventricular ectopy (trigger). Perhaps a consequence of mechanical traction exerted by the prolapsing leaflet, the fibrosis is most often localized to the posterior base of the left ventricle or the papillary muscles, which are most susceptible to the mechanical stretch forces exerted by the billowing leaflets [3].

The prolapsing mitral valve exercises traction on the papillary muscles, activating the local stretch receptors and causing membrane depolarization of the nerve endings with abnormal mechanoelectrical feedback to the central nervous system causing VT or VF [4].

A genetic background has been associated with MVP and SCD. MVP can be familial in 35–50% of cases [5]. High prevalence of MVP has been described in the following diseases: Trisomies 18, 13, 15, Marfan syndrome, Juvenile polyposis syndrome, Ehlers-Danlos syndrome, Loeys-Dietz, etc. [6]. A typical non-long QT associated mutation, in the SCN5A gene encoding the cardiac sodium channels, was found in case of SCD [7].

### Incidence of SCD in relation to MVP

The association between presence of MVP and occurrence of SCD has been estimated to be low. Meanwhile some studies and case reports have indicated that mitral valve prolapse may be a trigger for ventricular fibrillation. Thus, there is evidence for a significantly increased risk of SCD as well as risk for ventricular tachycardia in patients with MVP [2]. The prevalence of MVP is estimated at 1 to 3% in the population. This group has an approximately annual SCD risk of 0.2 to 1.9% [8].

### Risk factors

Ventricular tachycardia seems to be more common in female MVP patients, with some studies demonstrating that 70 to 90% of affected individuals are women [3]. The reason for this observation is unknown.

#### Electrocardiography

Premature ventricular contractions (PVC) are observed in the general population of MVP patients, with insignificantly difference between patients with and without SCD [9].

It had observed that the origin of PVC was majoretly the posterobasal segment of the LV. This constellation could be explained by a mechanical overload of the left ventricle in this area due to the defective functioning mitral valve [10]. This observation reveals the mechanism of the SCD in MVP as a complex electromechanical entity. Further explorations are required to confirm these findings. Basso and al describe that the majority of patients (> 75%) with MVP-related SCD demonstrate characteristic T-wave abnormalities on the surface ECG with biphasic or inverted T-waves in the inferior leads (II, III, aVF)(11). In this case, T-wave abnormalities are found in the inferior leads (III, aVF), recurrent PVC was recognized.

#### Cardiac imaging

The echocardiography allows a precise evaluation of the mitral valve. In addition to estimating the severity of mitral regurgitation and its mechanism, the echocardiography permits screening of risk parameters for sudden cardiac death due to MVP.

A possible signal of a high SCD-risk is a spiked high velocity signal across the lateral mitral annulus on echocardiography (“Pickelhaube sign”). This echocardiographic sign is more likely to be present in patients with (67%) than without (22%) ventricular arrhythmias.

A distinctive spiked signal (≥16 cm/s) appears to be a signal of a significant elevated SCD Risk [11].

A possible physiopathological explanation of this phenomenon is that the prolapsing leaflet tugs the posteromedial papillary muscle; consequently, the adjacent posterobasal area will be pressed toward the apex. Several studies have looked into the significance of this sign and its ability to detect patients with MVP and SCD risk. A study examined MVP patients simultaneously with CMR and echocardiography and has observed that only patients with Pickelhaube sign present LGE in the CMR. Therefore it was concluded that this sign an indicator of a malignant phenotype bileaflet mitral valve prolapse [12]. However, to confirm this finding, further investigation is needed.

Focal or diffuse myocardial fibrosis has been linked to ventricular arrhythmia and/or sudden cardiac arrest. Speckle-tracking echocardiography allows an assessment of left ventricular mechanical dispersion, which is an indicator of heterogeneity of ventricular contraction. MVP patients had significantly higher mechanical dispersion compared with controls despite similar LV ejection fraction, according to a study exploring MVP-patients with documented ventricular arrhythmia and MVP-patients without significant arrhythmia [13].

Furthermore, mitral annular disjunction (MAD), recently described entity, is characterized by detachment of the roots of the annulus from the ventricular myocardium to which it would normally be attached [14]. Several studies underline the correlation between MAD and SCD. However, the threshold limit (distance) of MAD that was associated with an increased risk SCD is controversial. A disjunction length of > 8.5 mm was detected by 67% of the patients who developed nonsustained VT on Holter monitoring [15]. 92% of morphologically typical MVP showed MAD, this was the result of a pathological study of 75 on 900 hearts from adult autopsies [16].

An interesting finding has been examined by a study; MAD might be considered as an independent risk factor for SCD, apart from the presence of a MVP. 22% of patients who suffered VT do not express any mitral valve anomaly [17].

Cardiac magnetic resonance (CMR) imaging is a noninvasive and powerful technique, with the ability to characterize myocardial tissue architecture and asses myocardial abnormalities. In addition, the CMR provides in a case of MVP and aborted SCD or VT important information.

CMR imaging with particular focus on delayed gadolinium enhancement of posterobasal myocardium and of the papillary muscles not only helps establish the diagnosis of MVP, it may leads to a risk stratification [1]. Delayed gadolinium enhancement in the posterobasal area, due to endocardial friction, is an additional mechanism for ventricular arrhythmias [18]. In addition to echocardiography, CMR allows accurate measurement of MAD. In this case, several risk indicators has been recognized in the echocardiography and CMR.

### Treatment for arrhythmic mitral valve prolapse

Due to the lack of uniform recommendations, an individualized therapy from patient with MVP and symptomatic ventricular arrhythmias should be considered, relying on the expertise of the medical team in each center.

A study demonstrated that ablation is feasible in MVP patients with symptomatic, drug-refractory ventricular arrhythmias. Moreover. Ablation can reduce symptomatic PVBs and appropriate ICD shocks during follow-up [12].

Mitral valve reconstruction results in a reduction of the ventricular volume load. It relieves stretch on the papillary muscles und facilitates ventricular reverse remodeling. Mitral valve surgery was found to be beneficial in reducing ventricular arrhythmias in a few studies [12]. Interesting that almost only younger patients showed significant reduction in ventricular ectopy, suggesting that early surgical intervention could modify the progress of the electrophysiologic substrate [12, 19].

Furthermore, a recent study shows that over a median follow-up of 9 years, 33% of patients developed hemodynamically significant VT/VF after their initial ablation and underwent placement of an implantable cardioverter defibrillator (ICD). This could indicate the progressive characteristics of the arrhythmia in patients with MVP [20].

The subsequent ICD-control after 3 weeks did not reveal any arrhythmia. We detected at the 3 months ICD-control a ventricular tachycardia. The VT was detected 3 days after the valve reconstruction. The subsequent ICD-control after 6 weeks did not reveal any arrhythmia. We decided to take a wait-and-see approach based on the preliminary studies on the suppressive effect of mitral valve reconstruction on VT, and the lack of rhythmogenic events in the subsequent ICD-controls. We envisaged ablation in case of VT-recurrence.

There is no clear recommendation for a primary prophylactic ICD implantation in patients with MVP and high risk for SCD. Neither the American Heart Association nor the European Society of Cardiology guidelines for ventricular arrhythmias and SCD have specific recommendations for the risk stratification of SCD in MVP. An electrophysiological examination should be considered to clarify the ICD indication. Some center consider implantation of an ICD if sustained monomorphic VT can be induced with up to 3 ventricular extrastimuli or sustained polymorphic VT can be induced with either 1 or 2 ventricular extrastimuli [21].

Due to the lack of prospective studies on this issue, the primary prophylactic ICD implantation remains an individual case decision.

## Conclusion

MVP is an underdiagnosed entity and is often encountered in asymptomatic individuals. Fibrosis of the papillary muscles and the posterobasal LV segment represents the structural substrate for the development of ventricular arrhythmias. Further more complex mechanisms for the occurrence of VT are reported.

Electrocardiographic, echocardiographic and MR-morphological risk features were identified and must be considered for patient stratification. In view of the new studies that have underlined the role of genetic substrate in MVP, genetic testing to determine high-risk individuals will be probably onducted in the future.

The current literature regarding the role of mitral valve repair/replacement in reducing ventricular arrhythmias is mixed and comes from isolated case reports or single-center experiences with small sample sizes. The role of electrophysiological exploration and ablation remained controversial. Currently, there are insufficient data supporting the role of primary prophylactic ICD implantation in patients with MVP and high-risk features.

In the case of our patient, ICD implantation was performed for secondary SCD prophylaxis.

From our point of view, MVP patients are indeed exposed to life-threatening arrhythmia, even SCD, therefore standardized treatment, focusing on primary and secondary prophylaxis is required. In case of unexplained life-threatening ventricular arrhythmias, we do believe that MVP must be considered and screened.

## Data Availability

The datasets used and/or analysed during the current study are available from the corresponding author on reasonable request.

## Abbreviations

ARVC: Arrhythmogenic right ventricular cardiomyopathy
PML: Posterior mitral leaflet
AML: Anterior mitral leaflet
MAD: Mitral annulus disjunction
CMR: Cardiovascular magnetic resonance imaging
MVP: Mitral valve prolapse
SCD: Sudden cardia death
TEE: Transesophageal echocardiogram
VT: Ventricular tachycardia
VF: Ventricular fibrillation
ICD: Implantable cardioverter-defibrillator
PM: Papillary muscle
